# Trichlorido[(meth­yl{2-[meth­yl(2-pyridyl­meth­yl)amino]eth­yl}amino)acetonitrile]iron(III) methanol hemisolvate

**DOI:** 10.1107/S160053680904375X

**Published:** 2009-10-28

**Authors:** Anne Nielsen, Christine J. McKenzie, Andrew D. Bond

**Affiliations:** a University of Southern Denmark, Department of Physics and Chemistry, Campusvej 55, 5230 Odense M, Denmark

## Abstract

The title compound, [FeCl_3_(C_12_H_18_N_4_)]·0.5CH_3_OH, contains an Fe^III^ ion in a distorted octa­hedral coordination environment. The neutral *N*,*N*′,*N*′′-tridentate ligand adopts a *fac* coordination mode, and chloride ligands lie *trans* to each of the three coordinated N atoms. In the crystal, the complexes form columns extending parallel to the approximate local threefold axes of the FeN_3_Cl_3_ octa­hedra, and the columns are arranged so that the uncoordinated nitrile groups align in an anti­parallel manner and the pyridyl rings form offset face-to-face arrangements [inter­planar separations = 2.95 (1) and 3.11 (1) Å; centroid–centroid distances = 5.31 (1) and 4.92 (1) Å]. The methanol solvent mol­ecule is disordered about a twofold rotation axis.

## Related literature

For structures of similar Fe^III^ complexes, see: Cowdell *et al.* (2004 ▶); Sundaravel *et al.* (2008 ▶); Velusamy *et al.* (2005 ▶).
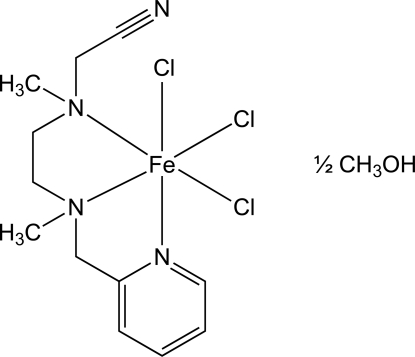


## Experimental

### 

#### Crystal data

[FeCl_3_(C_12_H_18_N_4_)]·0.5CH_4_O
*M* *_r_* = 396.53
Monoclinic, 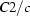

*a* = 34.243 (2) Å
*b* = 7.1331 (5) Å
*c* = 15.4835 (11) Å
β = 116.733 (3)°
*V* = 3377.8 (4) Å^3^
*Z* = 8
Mo *K*α radiation
μ = 1.37 mm^−1^
*T* = 180 K
0.18 × 0.10 × 0.10 mm

#### Data collection

Bruker–Nonius X8 APEXII CCD diffractometer
Absorption correction: multi-scan (*SADABS*; Bruker, 2004 ▶) *T*_min_ = 0.744, *T*_max_ = 0.875
38084 measured reflections
2937 independent reflections
2033 reflections with *I* > 2σ(*I*)
*R*_int_ = 0.066

#### Refinement

*R*[*F* ^2^ > 2σ(*F* ^2^)] = 0.040
*wR*(*F* ^2^) = 0.105
*S* = 1.07
2937 reflections
196 parameters
H-atom parameters constrained
Δρ_max_ = 0.48 e Å^−3^
Δρ_min_ = −0.45 e Å^−3^

### 

Data collection: *APEX2* (Bruker, 2004 ▶); cell refinement: *SAINT* (Bruker, 2004 ▶); data reduction: *SAINT*; program(s) used to solve structure: *SHELXTL* (Sheldrick, 2008 ▶); program(s) used to refine structure: *SHELXTL*; molecular graphics: *SHELXTL*; software used to prepare material for publication: *SHELXTL*.

## Supplementary Material

Crystal structure: contains datablocks global, I. DOI: 10.1107/S160053680904375X/hb5138sup1.cif

Structure factors: contains datablocks I. DOI: 10.1107/S160053680904375X/hb5138Isup2.hkl

Additional supplementary materials:  crystallographic information; 3D view; checkCIF report

## Figures and Tables

**Table 1:** Selected bond lengths (Å)

Fe1—N1	2.186 (3)
Fe1—N2	2.235 (3)
Fe1—N3	2.330 (3)
Fe1—Cl1	2.2873 (11)
Fe1—Cl2	2.2908 (11)
Fe1—Cl3	2.3284 (11)

**Fig. 1.**
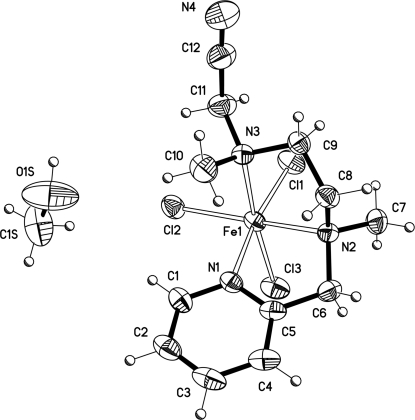
The molecular structure of (I) with displacement ellipsoids shown at 50% probability for non-H atoms. The methanol molecule (C1S—O1S) is disordered around a 2-fold rotation axis.

**Fig. 2.**
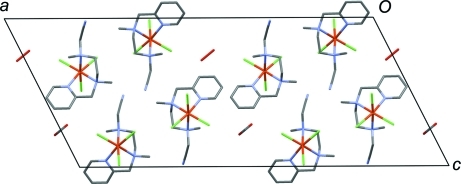
Unit-cell contents of (I) projected along the b axis, which corresponds to the stacking directions of the "columns" referred to in the Abstract.

## supplementary crystallographic information

### Comment

The ligand *N*,*N*'-dimethyl-*N*-(2-pyridylmethyl)ethylendiamine- *N*'-acetonitrile was prepared as a by-product during synthesis of *N*,*N*'-dimethyl-*N*-(2-pyridylmethyl)ethylendiamine- *N*'-acetic acid, as a result of contamination of the reagent bromoacetic acid with bromoacetonitrile.

### Experimental

The ligand synthesis was undertaken in three steps:

(i) Picolinal (2.50 ml, 24 mmol) and *N*,*N*'-dimethylethylenediamine (2.22 ml, 24 mmol) in dry diethylether (20 ml) were stirred overnight under CaCl_2_ protection. The solvent was removed under reduced pressure to leave 1,3-dimethyl-2-(2-pyridylmethyl)imidazolidine as a thin yellow oil (3.9 g, yield 92%).

(ii) NaBH_3_CN (1.3925 g, 22 mmol) and CF_3_COOH (3.365 ml, 44 mmol) were added in small portions [CAUTION: possible formation of HCN!] to 1,3-dimethyl-2-(2-pyridylmethyl)imidazolidine (3.8944 g, 22 mmol) in methanol (80 ml) and the reaction mixture was stirred overnight under CaCl_2_ protection. NaOH (85 ml of a 4 *M* aqueous solution) was added. The reaction mixture was stirred overnight and extracted with CHCl_2_ (3 × 20 ml), then the organic phase was dried over Na_2_SO_4_ and filtered. The filtrate was evaporated *in vacuo* to leave *N*,*N*'-dimethyl-*N*-(2-pyridylmethyl)ethylenediamine as a thin yellow oil (3.2 g, yield 81%).

(iii) A mixture of *N*,*N*'-dimethyl-*N*-(2-pyridylmethyl)ethylenediamine (3.1602 g, 18 mmol), bromoacetic acid (2.4499 g, 18 mmol) and triethylamine (2.444 ml, 18 mmol) in absolute ethanol (10 ml) was heated overnight under reflux and N_2_. The solvent was removed under reduced pressure, then the residue was re-dissolved in water, adjusted to pH 8 with conc. NaOH and washed with CH_2_Cl_2_ (3 × 15 ml). The aqueous phase was adjusted to pH 4 with conc. HCl then evaporated *in vacuo* to leave a mixture of *N*,*N*'-dimethyl-*N*-(2-pyridylmethyl)ethylendiamine- *N*'-acetonitrile (*L*) and *N*,*N*'-dimethyl-*N*-(2-pyridylmethyl)ethylendiamine- *N*'-acetic acid as a brown oil (6.0 g, yield 139% due to impurities of triethylammonium bromide).

The title compound was then prepared as follows:

Anhydrous FeCl_3_ (15.8 mg, 0.097 mmol) was added to the mixed ligand product from above (23.3 mg, 0.098 mmol) in methanol (1.75 ml), and a few yellow blocks of (I) were deposited overnight.

### Refinement

H atoms bound to C atoms were placed in idealized positions with C—H = 0.95–0.99 Å and refined as riding with *U*_iso_(H) = 1.2 or 1.5*U*_eq_(C). The methanol molecule is disordered around a 2-fold rotation axis and all of its atoms have site occupancy factor 0.5. The H atom of the OH group was placed along the O1S—Cl3^i^ vector [symmetry code (i): *x*, 1 - *y*, 1/2 + *z*], with O—H = 0.85 Å and refined as riding with *U*_iso_(H) = 1.5*U*_eq_(O).

### Crystal data

**Table:**

[FeCl_3_(C_12_H_18_N_4_)]·0.5CH_4_O	*F*(000) = 1632
*M**_r_* = 396.53	*D*_x_ = 1.559 Mg m^−^^3^
Monoclinic, *C*2/*c*	Mo *K*α radiation, λ = 0.71073 Å
Hall symbol: -C 2yc	Cell parameters from 5350 reflections
*a* = 34.243 (2) Å	θ = 2.6–21.6°
*b* = 7.1331 (5) Å	µ = 1.37 mm^−^^1^
*c* = 15.4835 (11) Å	*T* = 180 K
β = 116.733 (3)°	Block, yellow
*V* = 3377.8 (4) Å^3^	0.18 × 0.10 × 0.10 mm
*Z* = 8	

### Data collection

**Table:**

Bruker–Nonius X8 APEXII CCD diffractometer	2937 independent reflections
Radiation source: fine-focus sealed tube	2033 reflections with *I* > 2σ(*I*)
graphite	*R*_int_ = 0.066
Thin–slice ω and φ scans	θ_max_ = 25.0°, θ_min_ = 3.7°
Absorption correction: multi-scan (*SADABS*; Bruker, 2004)	*h* = −37→40
*T*_min_ = 0.744, *T*_max_ = 0.875	*k* = −8→8
38084 measured reflections	*l* = −18→18

### Refinement

**Table:**

Refinement on *F*^2^	Primary atom site location: structure-invariant direct methods
Least-squares matrix: full	Secondary atom site location: difference Fourier map
*R*[*F*^2^ > 2σ(*F*^2^)] = 0.040	Hydrogen site location: inferred from neighbouring sites
*wR*(*F*^2^) = 0.105	H-atom parameters constrained
*S* = 1.07	*w* = 1/[σ^2^(*F*_o_^2^) + (0.0478*P*)^2^ + 6.352*P*] where *P* = (*F*_o_^2^ + 2*F*_c_^2^)/3
2937 reflections	(Δ/σ)_max_ < 0.001
196 parameters	Δρ_max_ = 0.48 e Å^−^^3^
0 restraints	Δρ_min_ = −0.45 e Å^−^^3^

### Special details

**Table:**

Geometry. All e.s.d.'s (except the e.s.d. in the dihedral angle between two l.s. planes) are estimated using the full covariance matrix. The cell e.s.d.'s are taken into account individually in the estimation of e.s.d.'s in distances, angles and torsion angles; correlations between e.s.d.'s in cell parameters are only used when they are defined by crystal symmetry. An approximate (isotropic) treatment of cell e.s.d.'s is used for estimating e.s.d.'s involving l.s. planes.
Refinement. Refinement of *F*^2^ against ALL reflections. The weighted *R*-factor *wR* and goodness of fit *S* are based on *F*^2^, conventional *R*-factors *R* are based on *F*, with *F* set to zero for negative *F*^2^. The threshold expression of *F*^2^ > σ(*F*^2^) is used only for calculating *R*-factors(gt) *etc*. and is not relevant to the choice of reflections for refinement. *R*-factors based on *F*^2^ are statistically about twice as large as those based on *F*, and *R*- factors based on ALL data will be even larger.

### Fractional atomic coordinates and isotropic or equivalent isotropic displacement parameters (Å^2^)

**Table:**

	*x*	*y*	*z*	*U*_iso_*/*U*_eq_	Occ. (<1)
Fe1	0.132445 (17)	0.50145 (7)	0.14178 (4)	0.02864 (18)	
Cl1	0.19634 (3)	0.65429 (14)	0.23597 (8)	0.0446 (3)	
Cl2	0.09339 (3)	0.60710 (15)	0.21941 (7)	0.0419 (3)	
Cl3	0.10936 (3)	0.71324 (14)	0.01386 (7)	0.0449 (3)	
N1	0.07912 (9)	0.3232 (4)	0.0431 (2)	0.0303 (7)	
N2	0.16333 (9)	0.3137 (4)	0.0744 (2)	0.0301 (7)	
N3	0.15694 (10)	0.2550 (4)	0.2523 (2)	0.0310 (7)	
C1	0.03944 (12)	0.3100 (5)	0.0400 (3)	0.0362 (10)	
H1A	0.0349	0.3647	0.0907	0.043*	
C2	0.00547 (13)	0.2210 (6)	−0.0332 (3)	0.0425 (11)	
H2A	−0.0225	0.2177	−0.0343	0.051*	
C3	0.01180 (14)	0.1367 (6)	−0.1051 (3)	0.0476 (11)	
H3A	−0.0115	0.0715	−0.1557	0.057*	
C4	0.05248 (14)	0.1471 (6)	−0.1034 (3)	0.0439 (11)	
H4A	0.0576	0.0897	−0.1527	0.053*	
C5	0.08561 (12)	0.2433 (5)	−0.0281 (3)	0.0334 (9)	
C6	0.12956 (13)	0.2725 (6)	−0.0253 (3)	0.0373 (10)	
H6A	0.1379	0.1584	−0.0494	0.045*	
H6B	0.1279	0.3779	−0.0684	0.045*	
C7	0.20174 (12)	0.3989 (6)	0.0690 (3)	0.0399 (10)	
H7A	0.2138	0.3098	0.0392	0.060*	
H7B	0.2241	0.4296	0.1344	0.060*	
H7C	0.1927	0.5134	0.0300	0.060*	
C8	0.17688 (12)	0.1338 (5)	0.1299 (3)	0.0339 (9)	
H8A	0.2007	0.0754	0.1197	0.041*	
H8B	0.1518	0.0460	0.1058	0.041*	
C9	0.19227 (12)	0.1686 (5)	0.2357 (3)	0.0310 (9)	
H9A	0.2012	0.0485	0.2714	0.037*	
H9B	0.2180	0.2528	0.2604	0.037*	
C10	0.12260 (14)	0.1127 (6)	0.2399 (3)	0.0462 (11)	
H10A	0.1354	0.0147	0.2891	0.069*	
H10B	0.1114	0.0564	0.1754	0.069*	
H10C	0.0986	0.1739	0.2471	0.069*	
C11	0.17554 (14)	0.3223 (6)	0.3534 (3)	0.0466 (11)	
H11A	0.1968	0.4236	0.3625	0.056*	
H11B	0.1518	0.3759	0.3655	0.056*	
C12	0.19758 (15)	0.1724 (7)	0.4245 (3)	0.0495 (12)	
N4	0.21605 (14)	0.0568 (6)	0.4803 (3)	0.0660 (12)	
C1S	0.0000	0.3867 (12)	0.2500	0.081 (2)	
H1S1	−0.0264	0.3589	0.1902	0.122*	0.50
H1S2	0.0177	0.4799	0.2369	0.122*	0.50
H1S3	−0.0084	0.4363	0.2983	0.122*	0.50
O1S	0.0231 (3)	0.2310 (11)	0.2834 (5)	0.103 (3)	0.50
H1S	0.0438	0.2424	0.3402	0.155*	0.50

### Atomic displacement parameters (Å^2^)

**Table:**

	*U*^11^	*U*^22^	*U*^33^	*U*^12^	*U*^13^	*U*^23^
Fe1	0.0251 (3)	0.0238 (3)	0.0325 (3)	−0.0011 (2)	0.0090 (2)	−0.0001 (2)
Cl1	0.0361 (6)	0.0348 (6)	0.0520 (7)	−0.0047 (5)	0.0100 (5)	−0.0027 (5)
Cl2	0.0341 (6)	0.0442 (6)	0.0442 (6)	0.0051 (5)	0.0150 (5)	−0.0080 (5)
Cl3	0.0434 (6)	0.0320 (6)	0.0460 (6)	−0.0021 (5)	0.0083 (5)	0.0112 (5)
N1	0.0254 (18)	0.0280 (18)	0.0329 (18)	0.0023 (14)	0.0090 (14)	0.0036 (14)
N2	0.0262 (18)	0.0366 (19)	0.0250 (17)	−0.0030 (14)	0.0095 (14)	−0.0019 (14)
N3	0.0283 (18)	0.0358 (19)	0.0262 (17)	−0.0009 (14)	0.0098 (15)	−0.0019 (14)
C1	0.028 (2)	0.033 (2)	0.045 (2)	−0.0015 (18)	0.014 (2)	0.0012 (19)
C2	0.029 (2)	0.040 (2)	0.048 (3)	0.0003 (19)	0.008 (2)	0.013 (2)
C3	0.033 (3)	0.046 (3)	0.042 (3)	−0.007 (2)	−0.002 (2)	0.004 (2)
C4	0.044 (3)	0.045 (3)	0.029 (2)	−0.004 (2)	0.0046 (19)	−0.0008 (19)
C5	0.032 (2)	0.033 (2)	0.027 (2)	−0.0023 (17)	0.0048 (19)	0.0036 (18)
C6	0.038 (2)	0.046 (2)	0.027 (2)	−0.0031 (19)	0.0134 (19)	−0.0022 (18)
C7	0.035 (2)	0.048 (3)	0.042 (2)	−0.006 (2)	0.022 (2)	0.000 (2)
C8	0.030 (2)	0.034 (2)	0.035 (2)	0.0039 (18)	0.0116 (18)	−0.0020 (18)
C9	0.029 (2)	0.028 (2)	0.036 (2)	0.0025 (17)	0.0140 (18)	0.0016 (17)
C10	0.042 (3)	0.042 (3)	0.055 (3)	−0.002 (2)	0.022 (2)	0.016 (2)
C11	0.051 (3)	0.049 (3)	0.034 (2)	0.011 (2)	0.014 (2)	−0.002 (2)
C12	0.056 (3)	0.052 (3)	0.041 (3)	0.004 (2)	0.022 (2)	0.005 (2)
N4	0.071 (3)	0.071 (3)	0.054 (3)	0.007 (2)	0.027 (2)	0.017 (2)
C1S	0.073 (6)	0.071 (6)	0.119 (7)	0.000	0.061 (5)	0.000
O1S	0.121 (7)	0.052 (5)	0.074 (6)	0.008 (5)	−0.012 (5)	0.008 (4)

### Geometric parameters (Å, °)

**Table:**

Fe1—N1	2.186 (3)	C6—H6A	0.990
Fe1—N2	2.235 (3)	C6—H6B	0.990
Fe1—N3	2.330 (3)	C7—H7A	0.980
Fe1—Cl1	2.2873 (11)	C7—H7B	0.980
Fe1—Cl2	2.2908 (11)	C7—H7C	0.980
Fe1—Cl3	2.3284 (11)	C8—C9	1.500 (5)
N1—C1	1.341 (5)	C8—H8A	0.990
N1—C5	1.345 (5)	C8—H8B	0.990
N2—C6	1.484 (5)	C9—H9A	0.990
N2—C7	1.484 (4)	C9—H9B	0.990
N2—C8	1.497 (5)	C10—H10A	0.980
N3—C9	1.479 (4)	C10—H10B	0.980
N3—C11	1.480 (5)	C10—H10C	0.980
N3—C10	1.499 (5)	C11—C12	1.476 (6)
C1—C2	1.362 (5)	C11—H11A	0.990
C1—H1A	0.950	C11—H11B	0.990
C2—C3	1.365 (6)	C12—N4	1.155 (5)
C2—H2A	0.950	C1S—O1S	1.326 (9)
C3—C4	1.383 (6)	C1S—H1S1	0.980
C3—H3A	0.950	C1S—H1S2	0.980
C4—C5	1.388 (5)	C1S—H1S3	0.980
C4—H4A	0.950	O1S—O1S^i^	1.449 (16)
C5—C6	1.501 (5)	O1S—H1S	0.850
			
N1—Fe1—N2	75.33 (11)	N2—C6—H6A	109.4
N1—Fe1—Cl1	169.08 (9)	C5—C6—H6A	109.4
N2—Fe1—Cl1	93.76 (8)	N2—C6—H6B	109.4
N1—Fe1—Cl2	93.27 (8)	C5—C6—H6B	109.4
N2—Fe1—Cl2	162.33 (8)	H6A—C6—H6B	108.0
Cl1—Fe1—Cl2	97.20 (4)	N2—C7—H7A	109.5
N1—Fe1—Cl3	85.73 (8)	N2—C7—H7B	109.5
N2—Fe1—Cl3	92.44 (8)	H7A—C7—H7B	109.5
Cl1—Fe1—Cl3	95.42 (4)	N2—C7—H7C	109.5
Cl2—Fe1—Cl3	100.25 (4)	H7A—C7—H7C	109.5
N1—Fe1—N3	89.21 (10)	H7B—C7—H7C	109.5
N2—Fe1—N3	78.53 (10)	N2—C8—C9	110.6 (3)
Cl1—Fe1—N3	88.06 (8)	N2—C8—H8A	109.5
Cl2—Fe1—N3	88.00 (8)	C9—C8—H8A	109.5
Cl3—Fe1—N3	170.54 (8)	N2—C8—H8B	109.5
C1—N1—C5	118.7 (3)	C9—C8—H8B	109.5
C1—N1—Fe1	125.4 (3)	H8A—C8—H8B	108.1
C5—N1—Fe1	115.2 (2)	N3—C9—C8	110.2 (3)
C6—N2—C7	108.6 (3)	N3—C9—H9A	109.6
C6—N2—C8	108.8 (3)	C8—C9—H9A	109.6
C7—N2—C8	109.2 (3)	N3—C9—H9B	109.6
C6—N2—Fe1	107.1 (2)	C8—C9—H9B	109.6
C7—N2—Fe1	113.4 (2)	H9A—C9—H9B	108.1
C8—N2—Fe1	109.6 (2)	N3—C10—H10A	109.5
C9—N3—C11	108.8 (3)	N3—C10—H10B	109.5
C9—N3—C10	110.5 (3)	H10A—C10—H10B	109.5
C11—N3—C10	107.1 (3)	N3—C10—H10C	109.5
C9—N3—Fe1	103.9 (2)	H10A—C10—H10C	109.5
C11—N3—Fe1	111.9 (2)	H10B—C10—H10C	109.5
C10—N3—Fe1	114.5 (2)	C12—C11—N3	112.8 (3)
N1—C1—C2	122.3 (4)	C12—C11—H11A	109.0
N1—C1—H1A	118.9	N3—C11—H11A	109.0
C2—C1—H1A	118.9	C12—C11—H11B	109.0
C1—C2—C3	119.6 (4)	N3—C11—H11B	109.0
C1—C2—H2A	120.2	H11A—C11—H11B	107.8
C3—C2—H2A	120.2	N4—C12—C11	177.8 (5)
C2—C3—C4	119.3 (4)	O1S—C1S—H1S1	109.5
C2—C3—H3A	120.4	O1S—C1S—H1S2	109.5
C4—C3—H3A	120.4	H1S1—C1S—H1S2	109.5
C3—C4—C5	118.5 (4)	O1S—C1S—H1S3	109.5
C3—C4—H4A	120.7	H1S1—C1S—H1S3	109.5
C5—C4—H4A	120.7	H1S2—C1S—H1S3	109.5
N1—C5—C4	121.6 (4)	C1S—O1S—O1S^i^	56.9 (4)
N1—C5—C6	116.8 (3)	C1S—O1S—H1S	113.4
C4—C5—C6	121.5 (4)	O1S^i^—O1S—H1S	150.8
N2—C6—C5	111.1 (3)		
			
N2—Fe1—N1—C1	168.7 (3)	Cl2—Fe1—N3—C11	−51.8 (2)
Cl1—Fe1—N1—C1	165.9 (3)	N1—Fe1—N3—C10	−22.9 (3)
Cl2—Fe1—N1—C1	2.4 (3)	N2—Fe1—N3—C10	−98.1 (3)
Cl3—Fe1—N1—C1	−97.6 (3)	Cl1—Fe1—N3—C10	167.7 (3)
N3—Fe1—N1—C1	90.4 (3)	Cl2—Fe1—N3—C10	70.4 (2)
N2—Fe1—N1—C5	−21.2 (2)	C5—N1—C1—C2	−0.9 (5)
Cl1—Fe1—N1—C5	−24.1 (6)	Fe1—N1—C1—C2	168.8 (3)
Cl2—Fe1—N1—C5	172.5 (2)	N1—C1—C2—C3	2.0 (6)
Cl3—Fe1—N1—C5	72.4 (2)	C1—C2—C3—C4	−1.6 (6)
N3—Fe1—N1—C5	−99.6 (3)	C2—C3—C4—C5	0.1 (6)
N1—Fe1—N2—C6	32.1 (2)	C1—N1—C5—C4	−0.6 (5)
Cl1—Fe1—N2—C6	−148.5 (2)	Fe1—N1—C5—C4	−171.3 (3)
Cl2—Fe1—N2—C6	83.2 (3)	C1—N1—C5—C6	176.0 (3)
Cl3—Fe1—N2—C6	−52.9 (2)	Fe1—N1—C5—C6	5.3 (4)
N3—Fe1—N2—C6	124.3 (2)	C3—C4—C5—N1	1.0 (6)
N1—Fe1—N2—C7	151.8 (3)	C3—C4—C5—C6	−175.5 (4)
Cl1—Fe1—N2—C7	−28.7 (2)	C7—N2—C6—C5	−162.3 (3)
Cl2—Fe1—N2—C7	−157.0 (2)	C8—N2—C6—C5	78.9 (4)
Cl3—Fe1—N2—C7	66.9 (2)	Fe1—N2—C6—C5	−39.5 (4)
N3—Fe1—N2—C7	−116.0 (2)	N1—C5—C6—N2	24.1 (5)
N1—Fe1—N2—C8	−85.8 (2)	C4—C5—C6—N2	−159.3 (4)
Cl1—Fe1—N2—C8	93.7 (2)	C6—N2—C8—C9	−152.1 (3)
Cl2—Fe1—N2—C8	−34.7 (4)	C7—N2—C8—C9	89.5 (4)
Cl3—Fe1—N2—C8	−170.7 (2)	Fe1—N2—C8—C9	−35.3 (3)
N3—Fe1—N2—C8	6.4 (2)	C11—N3—C9—C8	−168.7 (3)
N1—Fe1—N3—C9	97.7 (2)	C10—N3—C9—C8	74.0 (4)
N2—Fe1—N3—C9	22.5 (2)	Fe1—N3—C9—C8	−49.3 (3)
Cl1—Fe1—N3—C9	−71.7 (2)	N2—C8—C9—N3	59.5 (4)
Cl2—Fe1—N3—C9	−169.0 (2)	C9—N3—C11—C12	−56.6 (4)
N1—Fe1—N3—C11	−145.1 (3)	C10—N3—C11—C12	62.9 (4)
N2—Fe1—N3—C11	139.7 (3)	Fe1—N3—C11—C12	−170.8 (3)
Cl1—Fe1—N3—C11	45.5 (2)		

Symmetry codes: (i) −*x*, *y*, −*z*+1/2.
